# lnc015013-CsMYB30-CsJAZ4/6 Module Co-Regulates JA Synthesis and Enhances Cold Hardiness in Tea Plants

**DOI:** 10.3390/ijms27114776

**Published:** 2026-05-26

**Authors:** Pingping Li, Zhaolan Han, Wei Huang, Huan Zhang, Xujun Zhu, Jie Jiang, Wanping Fang, Yuanchun Ma

**Affiliations:** 1 College of Horticulture, Nanjing Agricultural University, Nanjing 210095, China; 18788609273@163.com (P.L.); hanzl@njau.edu.cn (Z.H.); huangwei@stu.njau.edu.cn (W.H.); zhanghuan2131@163.com (H.Z.); zhuxujun@njau.edu.cn (X.Z.); jiangjie@njau.edu.cn (J.J.); fangwp@njau.edu.cn (W.F.); 2 Zunyi Academy of Agricultural Sciences, Zunyi 563000, China

**Keywords:** tea plant, Longjing 43, cold stress, jasmonic acid, long non-coding RNA, MYB, JAZ

## Abstract

Tea plants (*Camellia sinensis*) suffer growth limitations under cold stress. Jasmonic acid (JA) and long non-coding RNAs (lncRNAs) are involved in stress responses, yet how lncRNAs regulate JA-mediated cold tolerance remains unclear. Here, we identified an lncRNA, *lnc015013*, whose silencing compromised cold tolerance in tea plants, a phenotype rescued by exogenous methyl jasmonate (MeJA). Silencing *lnc015013* down-regulated *CsMYB30* and *CsJAZ4/6*, while its overexpression had opposite effects. Heterologous expression in *Arabidopsis thaliana* showed that *CsMYB30* enhanced cold resistance, whereas *CsJAZ4/6* suppressed it. Mechanistically, CsMYB30 repressed *CsJAZ4/6* promoter activity and physically interacted with CsJAZ4/6, with MeJA attenuating this interaction. These findings reveal that the lnc015013-CsMYB30-CsJAZ4/6 module regulates JA biosynthesis within the JA signaling pathway, providing a novel mechanism for cold adaptation in tea plants and a theoretical basis for molecular breeding.

## 1. Introduction

Tea plants, as an important cash crop widely grown worldwide, play a key role in agriculture. However, their growth and yield are strongly influenced by environmental factors, and cold stress is one of the common and difficult stress challenges during the growth of tea plants. Low-temperature environments cause lipid peroxidation of cell membranes, protein denaturation, and metabolic disorders, which seriously impede the normal development of tea plants and affect the yield [1,2,3]. Therefore, it is of great significance to understand the response mechanism of tea plants to cold stress.

Jasmonic acid (JA), as a crucial phytohormone, plays a key role in plant response to stress [4,5]. Jasmonic acid (JA) is a fatty acid derivative deeply involved in plant growth, development, metabolic regulation and stress responses [6]. Under low-temperature stress, jasmonic acid can regulate gene expression, activate the antioxidant system, and contribute to the accumulation of osmoregulatory substances, thus assisting plants in resisting low-temperature attack [7]. Wang et al. found that *SlWRKY50* enhances cold tolerance by controlling JA biosynthesis [8]; the physiological and biochemical analyses conducted by the group have also confirmed that exogenous MeJA application can effectively help tea plants to scavenge ROS and stabilize cell membranes under cold stress [9]. Although JA helps plants to resist cold, its fine molecular mechanism used to regulate the response of tea plants to cold stress still needs to be explored.

Along with the continuous progress of high-throughput sequencing technology, the regulatory value of long non-coding RNA (lncRNA) in response to plant stress has been increasingly noticed [10,11]. lncRNAs are more than 200 nucleotides in length, and although they do not code for proteins, they can widely penetrate into the regulation of gene expression by interacting with DNA, RNA, or proteins, by epigenetic modification, and by signal transduction [12,13]. lncRNAs are frequently involved in the response of plants to various adversities such as drought, salt stress and cold stress [14]. In the face of cold stress, they can regulate the expression of cold-resistant genes [15,16] to strengthen the cold resistance of plants, act as a “molecular sponge” that adsorbs miRNAs to regulate the expression of target genes [17], interact with proteins to influence their function and stability [18], or participate in chromatin remodeling to regulate the transcriptional activity of genes [19].

Nevertheless, the involvement of lncRNAs in JA-mediated cold stress responses in tea plants is unexplored. This study investigates the mechanistic role of lncRNAs in JA-regulated cold tolerance. Through RNA-seq analysis, we identified *lnc015013* and examined its expression dynamics alongside putative targets *CsJAZ4/6* and *CsMYB30* under low-temperature stress. Integrating transgenic phenotyping, biochemical assays, and physiological analyses, we reveal how *lnc015013* regulates *CsJAZ4/6* and *CsMYB30* to modulate JA biosynthesis. Our findings establish that the lnc015013-CsMYB30-CsJAZ4/6 complex co-regulates JA synthesis to enhance cold tolerance, providing novel theoretical insights and technical frameworks for cold-resistance breeding in tea plants.

## 2. Results

### 2.1. Transcriptome Analysis of MeJA Treatment on Tea Plants in Response to Low-Temperature Stress

**Figure 1:**
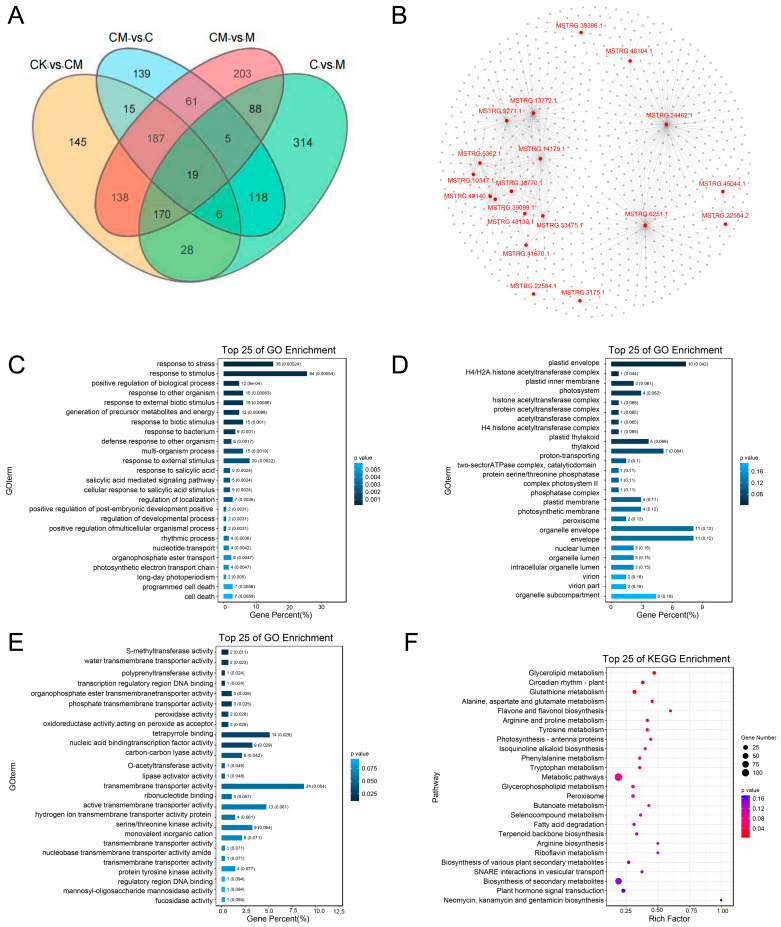
Transcriptome analysis of MeJA treatment on tea plants in response to low-temperature stress. (**A**) Screening of differentially expressed lncRNAs; (**B**) network map of lncRNAs with their target genes; (**C**) GO enrichment analysis of target genes in biological processes; (**D**) GO enrichment analysis of target genes in cellular components; (**E**) GO enrichment analysis of target genes in molecular functions; (**F**) KEGG enrichment analysis of target genes. *n* = 3 biological replicates per treatment for RNA-seq.

To characterize the lncRNAs of jasmonic acid (JA) in tea plants involved in the response to cold stress, we performed RNA-seq analysis on 12 tea plant new shoot samples ((control)1–3; (cold)1–3; (MeJA)1–3; (cold+MeJA)1–3) to analyze their targeting relationships and map the network, and Gene Ontology (GO) and Kyoto Encyclopedia of Genes and Genomes (KEGG) enrichment analyses were carried out on the target genes, obtaining different categories of significantly enriched entries and seven significantly enriched metabolic pathways that are closely related to plant stress tolerance. Nineteen lncRNAs that were differentially expressed in all four comparison groups were screened by Venn diagrams (Figure 1A). These lncRNAs regulated 1335 target genes through trans-activation (Figure 1B). GO enrichment analysis showed that these target genes had a significant role in “stress response” in biological processes, “plasma membrane” in cellular components, and “S-methyltransferase” in molecular functions (Figure 1C–E). KEGG pathway analysis further revealed seven significantly enriched metabolic pathways (Figure 1F). These pathways include glycerol ester metabolism, glutathione metabolism, and flavonoid biosynthesis, which are closely related to plant stress tolerance.

In summary, the RNA-seq analysis revealed that *lnc015013* and its target genes play significant roles in the JA signaling pathway and plant stress tolerance. The differentially expressed lncRNAs and their target genes provide valuable insights into the molecular mechanisms underlying the response of tea plants to low-temperature stress.

### 2.2. Identification of lnc015013 and Its Target Genes in Tea Plants

**Figure 2:**
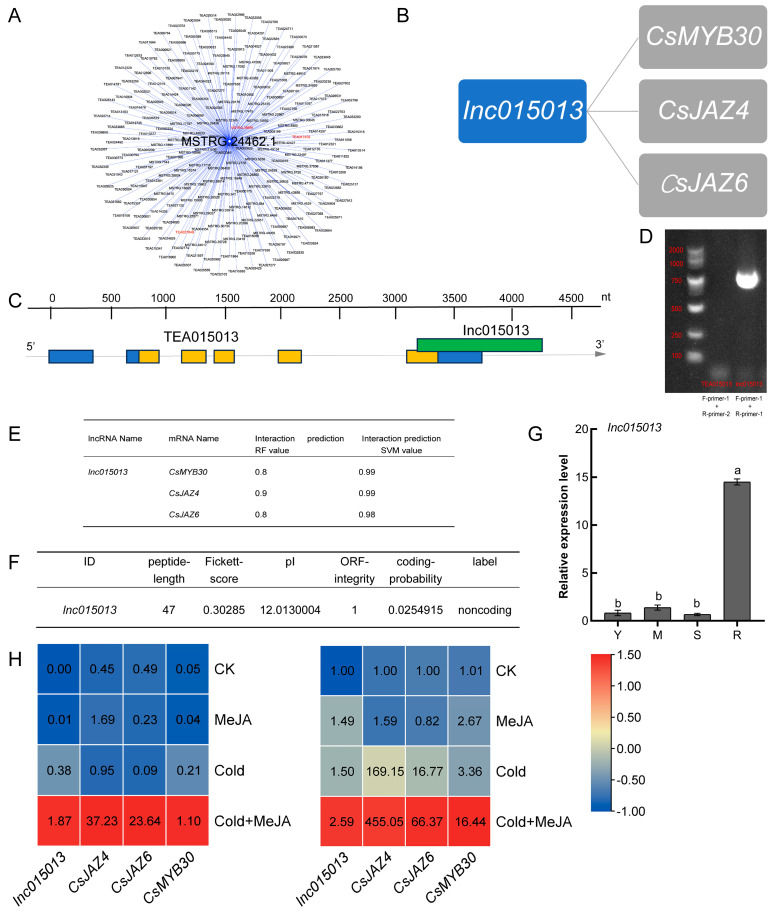
Positional identification of *lnc015013*. (**A**) Network diagram of *lnc015013* with its target genes; the red text in (**A**) represents the genes studied in this article.; (**B**) target genes of *lnc015013* in the JA signaling pathway; (**C**) positional identification of lnc015013; (**D**) gel electrophoresis identification of *lnc015013* and *TEA015013*; (**E**) RNA–protein interaction predictions for *lnc015013* and its target gene transcripts; (**F**) prediction of coding ability of *lnc015013*; (**G**) expression levels of *lnc015013* in different tissues of tea plants (*p* < 0.05). Note: Y: young leaf; M: mature leaf; S: stem; R: root; Different letters (a, b) above the bars indicate statistically significant differences (*p* < 0.05); (**H**) Expression levels of *lnc015013*, *CsMYB30*, *CsJAZ4* and *CsJAZ6* (*p* < 0.05). *n* = 3 biological replicates for expression data; *n* = 5 for root expression. Error bars represent SD.

From the 19 differentially expressed lncRNAs, we focused on *lnc015013*, which targets *CsJAZ4*, *CsJAZ6*, and *CsMYB30*, genes that play key roles in the JA signaling pathway (Figure 2A). Sequence analysis confirmed that *CsJAZ4* and *CsJAZ6* belonged to the JAZ family, whereas *CsMYB30* belonged to the R2R3-MYB family (Figure 2B). Polymerase chain reaction (PCR) amplification confirmed that *lnc015013* was a stand-alone transcript that was separated from the mRNA of *TEA015013* (Figure 2D). RNA–protein interaction predictions suggested potential binding between *lnc015013* and the transcripts of its target genes, although this requires experimental validation (Figure 2E). In addition, *lnc015013* was predicted to be incapable of encoding proteins (Figure 2F) and had the highest expression in roots (Figure 2G). By comparing the RNA-seq and quantitative polymerase chain reaction (qPCR) data, the expression patterns of *lnc015013*, *CsMYB30*, *CsJAZ4* and *CsJAZ6* were consistent under different treatments (Figure 2H), which verified the reliability of the transcriptome data.

### 2.3. Functional Validation of lnc015013 as a Positive Regulator of Jasmonic Acid-Mediated Cold Tolerance

**Figure 3:**
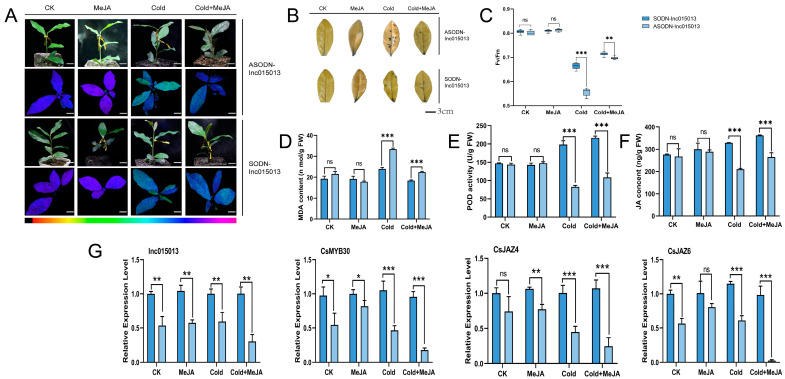
Silencing *lnc015013* tea plants in response to low-temperature stress. (**A**) Phenotypes under silencing *lnc015013* tea plant treatment, scale bar = 5 cm; (**B**) NBT staining map, scale bar = 3 cm; (**C**) chlorophyll fluorescence parameters Fv/Fm; (**D**) MDA content; (**E**) POD enzyme activity; (**F**) JA content; (**G**) silencing efficiency and expression of target genes (*n* = 10 seedlings per genotype for Fv/Fm; *n* = 3 biological replicates for MDA, POD, JA and qRT-PCR). Error bars represent SD. * *p* < 0.05, ** *p* < 0.01, *** *p* < 0.001, ns: not significant.

In this study, antisense oligonucleotide (AsODN) transient silencing technology was used to resolve the role of *lnc015013* in the cold tolerance of tea plants (Figure 3A). Visualization of the chlorophyll fluorescence parameter Fv/Fm (maximum photochemical efficiency of photosystem II) showed that after low-temperature stress, the Fv/Fm value of AsODN (*lnc015013* silenced) plants was 16.13% lower than that of the SODN control, indicating that silencing *lnc015013* exacerbated photoinhibition. Compared with their respective CK (normal) groups, Fv/Fm in SODN plants decreased by 17.79%, whereas in AsODN plants it decreased by 28.84%. Exogenous MeJA treatment significantly increased the Fv/Fm value by 6.06% compared with untreated AsODN plants under cold stress, suggesting that MeJA partially rescued the photosystem damage caused by *lnc015013* silencing (Figure 3C). NBT staining showed that the leaves of the silenced plants exhibited dark blue patches, indicating that the *lnc015013* inhibition significantly weakened the cold resistance of tea plants, while exogenous MeJA treatment could effectively restore the cold-resistant phenotype of leaves affected by low temperature (Figure 3B).

Analysis of the physiological indices of cold tolerance showed that under cold stress conditions, the MDA content of *lnc015013*-suppressed plants was elevated by 39.46% compared with the control (Figure 3D); peroxidase (POD) activity was reduced by 58.20% (Figure 3E); and JA concentration was decreased by 36.01% (Figure 3F). Relative to their own CK, the MDA content in SODN plants increased by 39.46% and in AsODN plants by 67.52%; POD activity decreased by 58.20% in SODN plants and by 54.30% in AsODN plants; and JA concentration decreased by 36.01% in SODN plants and by 60.17% in AsODN plants. Exogenous MeJA treatment further decreased the MDA content by 22.92% (Figure 3D); increased POD activity by 30.81% (Figure 3E); and increased JA concentration by 25.8% compared with untreated plants (Figure 3F).

The qRT-PCR results showed that silencing *lnc015013* decreased the expression of *CsMYB30*, *CsJAZ4* and *CsJAZ6*. The specific value changes were as follows: the expression of *CsMYB30* decreased by about 50%, the expression of *CsJAZ4* decreased by about 40%, and the expression of *CsJAZ6* decreased by about 50% (Figure 3G).

**Figure 4:**
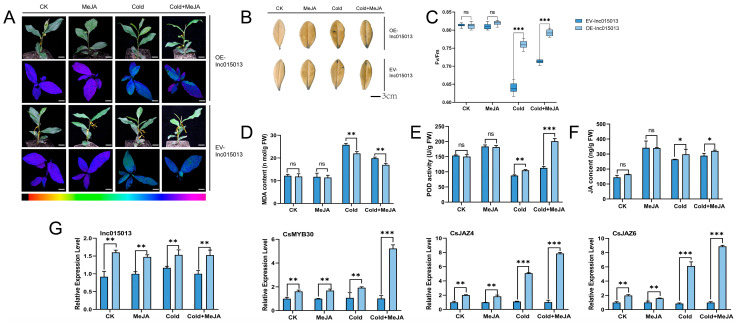
Response of overexpressed *lnc015013* tea plant to low-temperature stress. (**A**) Phenotypes under overexpression of *lnc015013* tea plant treatment, scale bar = 5 cm; (**B**) NBT staining plot, scale bar = 3 cm; (**C**) chlorophyll fluorescence parameter Fv/Fm; (**D**) MDA content; (**E**) POD enzyme activity; (**F**) JA content; and (**G**) expression of target genes (*n* = 10 seedlings per genotype for Fv/Fm; *n* = 3 biological replicates for MDA, POD, JA and qRT-PCR). Error bars represent SD. * *p* < 0.05, ** *p* < 0.01, *** *p* < 0.001, ns: not significant.

After cold stress treatment, the fluorescence intensity of tea plant leaves was significantly higher after *lnc015013* was overexpressed compared with the control (empty vector, EV), indicating that the degree of damage to tea plant leaves was less after overexpression; when the MeJA group was sprayed, the fluorescence intensity of transgenic *lnc015013* tea plants was significantly higher than that of the unsprayed MeJA group, which reduced the degree of damage to tea plant leaves caused by the low-temperature treatment (Figure 4A).

Visualization of the chlorophyll fluorescence parameter maximum photochemical efficiency of photosystem II (Fv/Fm) revealed that the Fv/Fm value of overexpressed *lnc015013* plants was increased by 18.75% compared with that of the empty vector (EV) control after low-temperature stress, and the Fv/Fm value of was further increased by 3.95% compared with that of the untreated plants after treatment with exogenous MeJA (Figure 4C). Compared with CK, Fv/Fm in EV plants decreased by 18.60%, whereas in OE plants it decreased by only 3.95%. NBT staining results showed that the leaves of the overexpressed plants stained lighter and did not have any blue patches, which indicated that the results of nitroblue tetrazolium (NBT) staining showed that the leaves of overexpressed plants had lighter staining and no blue patches, indicating that the heterologous expression of *lnc015013* significantly enhanced the cold resistance of tea plants, and the exogenous MeJA treatment could further alleviate the leaf damage caused by low temperature (Figure 4B).

The analysis of the physiological indicators of cold tolerance showed that under cold stress conditions, the MDA content of overexpressed *lnc015013* plants was reduced by 14.58% compared with the control (Figure 4D); POD activity was increased by 19.40% (Figure 4E); and JA concentration was elevated by 12.77% (Figure 4F). Relative to their own CK, the MDA content in EV plants increased by 14.61%, while in OE plants it increased by only 1.41%; POD activity decreased by 19.40% in EV plants but increased by 19.40% in OE plants; and JA concentration decreased by 12.78% in EV plants but increased by 12.78% in OE plants. After exogenous MeJA treatment, the MDA content was further reduced by 22.93% (Figure 4D); POD activity was increased by 91.87% (Figure 4E); and JA concentration was increased by 7.56% compared with untreated plants (Figure 4F).

The qRT-PCR results showed that overexpression of *lnc015013* increased the expression of *CsMYB30*, *CsJAZ4* and *CsJAZ6*. The specific value changes were as follows: the expression of *CsMYB30* increased about 1.5-fold, the expression of *CsJAZ4* increased about 2-fold, and the expression of *CsJAZ6* increased about 2-fold (Figure 4G).

**Figure 5:**
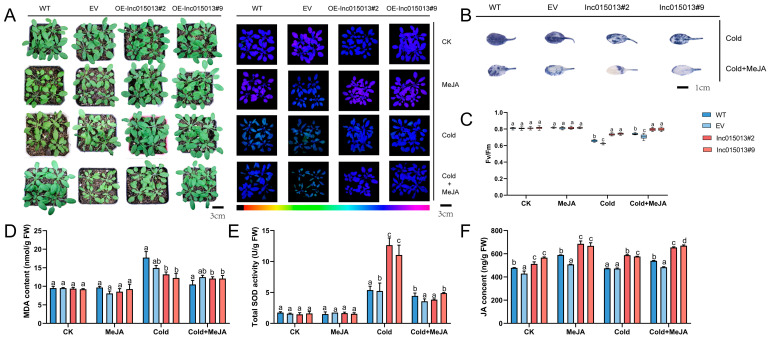
Response of *Arabidopsis thaliana* heterologously expressing *lnc015013* to low-temperature stress. (**A**) Phenotypes under heterologously expressed *lnc015013* Arabidopsis treatment, scale bar = 3 cm; (**B**) NBT staining plots, scale bar = 1 cm; (**C**) chlorophyll fluorescence parameter Fv/Fm; (**D**) MDA content; (**E**) SOD enzyme activity; (**F**) JA content (*n* = 10 seedlings per genotype for Fv/Fm; *n* = 3 biological replicates for MDA, SOD, JA). Error bars represent SD. Different letters (a, b, c, d) above the bars indicate statistically significant differences (*p* < 0.05).

In this study, we further determined the cold tolerance role of *lnc015013* in model plants by transforming its expression vector into *Arabidopsis thaliana*. Under normal conditions, no significant difference in growth was observed among wild-type (WT), empty vector (EV) and transgenic Arabidopsis. Under low-temperature stress, heterologous expression of *lnc015013* significantly enhanced cold tolerance (Figure 5A,B). Compared with their respective CK, the MDA content in WT and EV plants increased by 39.62%, whereas in OE-*lnc015013* plants it increased by only 17.62%. The OE line showed a 22.04% decrease in MDA compared with WT/EV (Figure 5D). SOD activity increased approximately 2-fold relative to CK in OE plants, whereas WT and EV plants showed only a 1.2-fold increase (Figure 5E). Fv/Fm relative to CK decreased by 17.79% in WT and 19.30% in EV but only by 5.02% in OE plants; OE plants had a 15.63% higher Fv/Fm than controls (Figure 5C). Endogenous JA content relative to CK decreased by 12.50% in WT and 11.71% in EV but increased by 22.93% in OE plants; OE plants had 22.93% higher JA than controls (Figure 5C). Exogenous MeJA treatment further enhanced these cold-tolerance-related indexes: MDA content was additionally reduced by 5.02%, Fv/Fm increased by 6.76%, and JA content increased by 13.78% (Figure 5C–F). This suggests that the MeJA treatment has a synergistic effect with heterologous expression of *lnc015013*, enhancing cold tolerance by strengthening the JA signaling pathway and antioxidant system.

In summary, *lnc015013* significantly enhanced cold tolerance in Arabidopsis by activating the JA signaling pathway and antioxidant defense system, and exogenous MeJA treatment further strengthened this effect.

### 2.4. Identification of CsMYB30 as a Positive Regulator of Jasmonic Acid-Mediated Cold Tolerance

**Figure 6:**
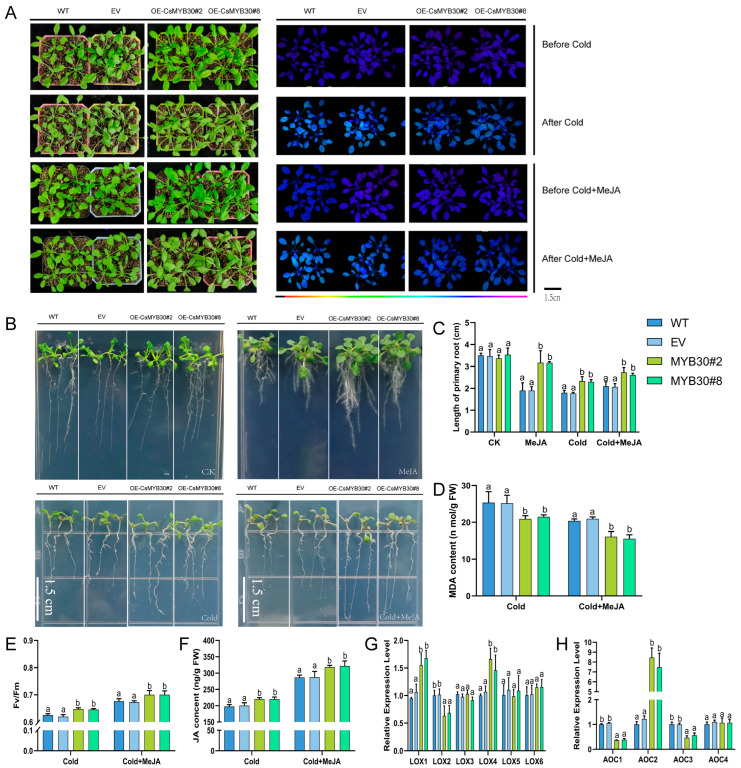
Heterologously expressed *CsMYB30* in *Arabidopsis thaliana* in response to low-temperature stress. (**A**) Length of primary roots under heterologously expressed *CsMYB30* Arabidopsis treatment, scale bar = 1.5 cm; (**B**) phenotypes under heterologously expressed *CsMYB30* Arabidopsis treatment, scale bar = 1.5 cm; (**C**) length of primary roots of Arabidopsis seedlings; (**D**) MDA content; (**E**) chlorophyll fluorescence parameter Fv/Fm; (**F**) JA content; (**G**) expression levels of genes related to jasmonate synthesis pathway; and (**H**) expression of target genes (*n* = 10 seedlings per genotype for root length and Fv/Fm; *n* = 3 biological replicates for MDA, JA and qRT-PCR). Error bars represent SD. Different letters (a, b) above the bars indicate statistically significant differences (*p* < 0.05).

To investigate the role of *CsMYB30* in cold resistance, this study was carried out to verify the function by constructing *Arabidopsis thaliana* lines heterologously expressing *CsMYB30* (heterologous expression lines, OE30#2/OE30#8). Phenotypic analysis showed that the primary roots of the overexpression (OE) lines were significantly longer than those of the wild type (WT) and the empty vector (EV) plants under low-temperature treatment, and the cold+MeJA treatment further alleviated the low-temperature-induced root growth inhibition (Figure 6A,C). After low-temperature stress, the OE strain showed less leaf drooping than the control and significantly higher values of chlorophyll fluorescence parameters Fv/Fm than WT/EV, and the cold+MeJA treatment enhanced this protective effect (Figure 6B,E). Physiological indexes showed that the malondialdehyde (MDA) content of the OE strain was significantly lower than that of the control at low temperature, while JA accumulation was significantly higher; exogenous MeJA treatment further reduced the MDA level and synergistically enhanced the JA content of the OE strain (Figure 6D,F). qRT-PCR analyses revealed that key genes for JA synthesis (*AtLOX1/4*, *AtAOC2*, and *AtOPR2*) were significantly up-regulated in the OE strain, whereas some of the degradation genes (*AtLOX2*, *AtAOC1/3*, *AtAOS*, etc.) were repressed, suggesting that *CsMYB30* maintains endogenous JA homeostasis by regulating the JA anabolic network. In summary, *CsMYB30* enhances the ability of plants to cope with low-temperature stress by positively regulating the JA biosynthesis pathway, and its heterologous expression significantly improves JA-mediated cold resistance (Figure 6G,H). This study provides a new basis for analyzing the molecular mechanism of cold resistance in plants.

### 2.5. CsJAZ4/6 Negatively Regulates Jasmonic Acid-Mediated Cold Tolerance

To determine the cold resistance function of *CsJAZ4/6*, their expression vectors were transformed into *Arabidopsis thaliana*. Under normal conditions, no significant differences in growth were observed between the transgenic lines and the wild-type (WT) or empty vector (EV) controls.

**Figure 7:**
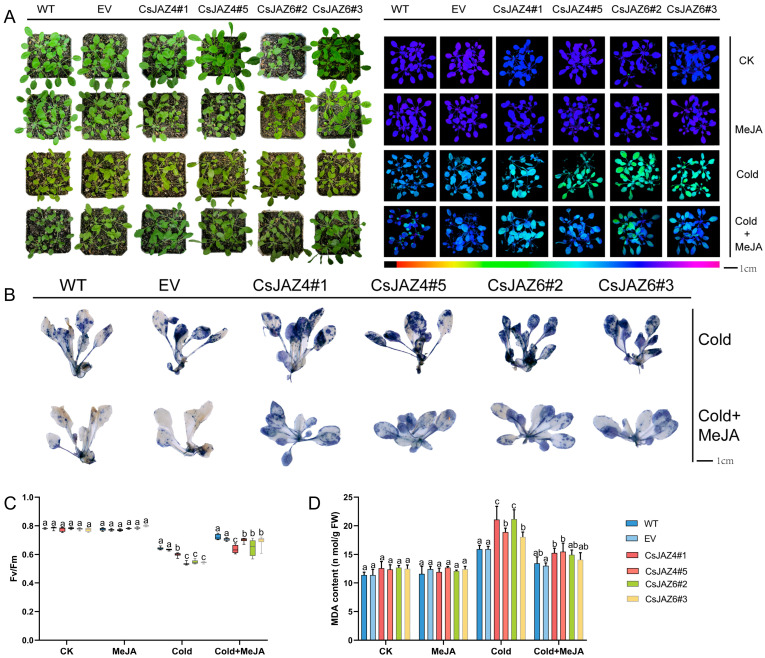
Response of Arabidopsis heterologously expressing CsJAZ4/6 to low-temperature stress. (**A**) Phenotypes under heterologously expressed CsJAZ4/6 Arabidopsis treatment, scale bar = 1 cm; (**B**) NBT staining plots, scale bar = 1 cm; (**C**) chlorophyll fluorescence parameters Fv/Fm; (**D**) MDA content. *n* = 10 seedlings per genotype for Fv/Fm; *n* = 3 biological replicates for MDA. Error bars represent SD. Different letters (a, b, c) above the bars indicate statistically significant differences (*p* < 0.05).

After cold stress, the maximum photochemical efficiency of photosystem II (Fv/Fm) decreased significantly. In WT and EV plants, Fv/Fm decreased by 17.79% and 19.30%, respectively. In lines heterologously expressing *CsJAZ4*, Fv/Fm decreased by 22.83% to 31.75%, and in lines heterologously expressing *CsJAZ6*, by 29.68% to 29.90% (Figure 7C). Exogenous MeJA treatment partially restored Fv/Fm under cold stress. In CsJAZ4#5, Fv/Fm increased by 31.09% compared with the untreated cold-stressed plants, and in CsJAZ6#2, it increased by 18.32% (Figure 7C).

Malondialdehyde (MDA) content increased markedly under cold stress. In WT and EV plants, MDA increased by 39.62%. In lines heterologously expressing *CsJAZ4*, MDA increased by 53.04% to 67.52%, and in lines heterologously expressing *CsJAZ6* by 44.84% to 66.97% relative to their respective CK groups. NBT staining also showed increased reactive oxygen species accumulation in the transgenic lines (Figure 7B,D). After the MeJA treatment, the MDA content decreased. In CsJAZ4#1, MDA decreased by 27.56% compared with the untreated cold-stressed plants (Figure 7D).

These results demonstrate that heterologous expression of *CsJAZ4/6* impairs low-temperature acclimation, exacerbates photosystem damage and oxidative stress, and thus negatively regulates cold tolerance. Exogenous MeJA alleviates the cold-induced injury but does not fully restore the cold-sensitive phenotype of lines heterologously expressing *CsJAZ4/6*. In conclusion, *CsJAZ4/6* acts as a negative regulator in the cold-response pathway, and its function is closely associated with the JA signaling pathway.

### 2.6. *CsMYB30* Can Bind the Promoters of CsJAZ4/6 and Repress Their Expression

**Figure 8:**
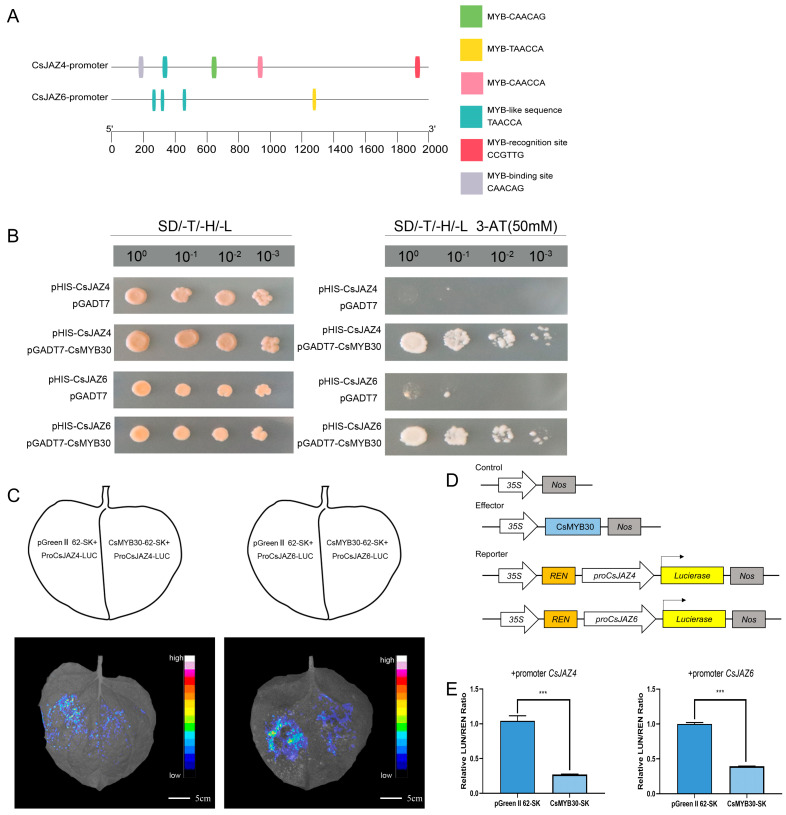
Validation of CsJAZ4/6 regulation by CsMYB30. (**A**) Predicted positions of promoter binding elements; (**B**) yeast one-hybrid assay result map; (**C**) in vivo imaging map of the dual luciferase assay, scale bar = 5 cm; (**D**) schematic diagram of the dual luciferase expression vector; (**E**) enzyme activity measurements of the dual luciferase assay (*n* = 3 independent yeast transformations for Y1H; *n* = 6 tobacco leaf injections for dual-luciferase). Error bars represent SD. *** *p* < 0.001.

To confirm the binding relationship between CsMYB30 and *CsJAZ4/6* promoters, yeast one-hybrid (Y1H) point-to-point experiments were performed. The results showed that the experimental groups AD-CsMYB30+pHis2-pro *CsJAZ4* and AD-CsMYB30+ pHis2-pro *CsJAZ6* could have colony growth on triple-deficient medium supplemented with 50 mM 3-AT, while the control group could not, suggesting that CsMYB30 can interact with the promoter fragments of *CsJAZ4/6* in this yeast one-hybrid assay (Figure 8B).

The effect of CsMYB30 versus *CsJAZ4/6* promoter activity was further analyzed using a dual-luciferase reporter system, with the CsMYB30 driven by the 35S promoter and the *CsJAZ4/6* promoter driving Luc reporter gene expression. The results showed that the Luc/Ren luciferase ratio in tobacco leaves co-injected with CsMYB30-62SK and the reporter gene vectors pro *CsJAZ4*: Luc/pro *CsJAZ6*: Luc was significantly lower than that in the negative control (pGreenII 62-SK empty+ reporter gene vector) (Figure 8C). In addition, we repeated the above assay in tobacco leaves and determined the fluorescence values of LUC and REN with a dual-luciferase detection kit. The results showed that the LUC/REN ratio in tobacco leaves supplemented with the CsMYB30-62SK plasmid was significantly lower than that of the empty load (Figure 8E). This indicates that the CsMYB30 transcription factor has the ability to repress *CsJAZ4/6* expression in tobacco leaves, consistent with the finding that CsMYB30 represses the activity of the *CsJAZ4/6* promoter.

### 2.7. *CsMYB30* and *CsJAZ4/6* Together Form a Key Regulatory Module

**Figure 9:**
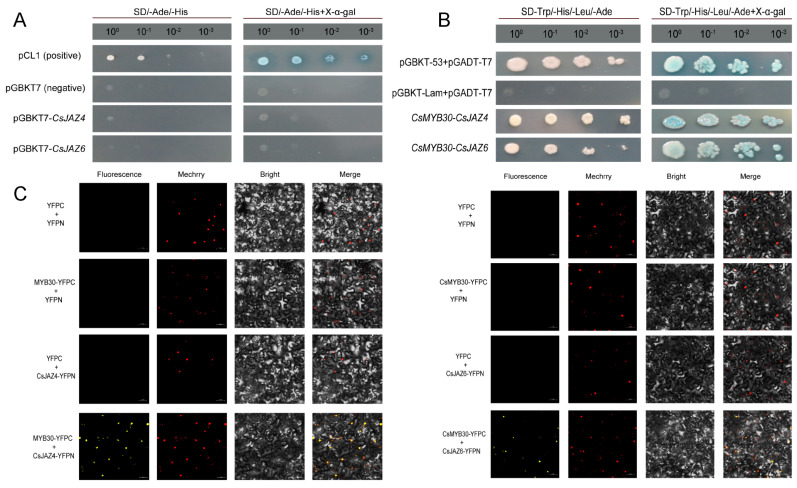

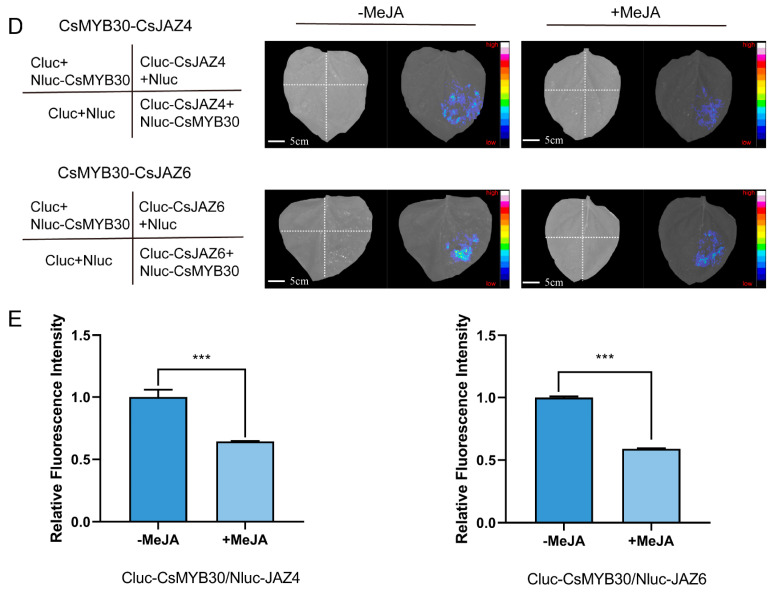
CsMYB30 interactions with CsJAZ4/6. (**A**) Transcriptional self-activation validation; (**B**) yeast two-hybrid validation; (**C**) bimolecular fluorescence complementation assay (BiFC), scale bar = 50 μm; (**D**) firefly luciferase fragment complementation (LCI) validation, scale bar = 5 cm; (**E**) fluorescence intensity measurement (*n* = 3 independent yeast colonies for Y2H; *n* = 10 cells for BiFC; *n* = 10 tobacco leaves for LCI). Error bars represent SD. *** *p* < 0.001.

To verify the interaction between CsMYB30 and CsJAZ4/6, yeast two-hybrid point-to-point experiments were performed. The experimental results showed that Y2H Gold yeast receptor strains co-transformed in the positive control and experimental groups were able to grow and show blue color on SD-Ade/-His/-Leu/-Trp-deficient medium coated with X-α-Gal, whereas yeast cells co-transfected with two negative controls could not grow on SD-Ade/-His/-Leu/-Trp-deficient medium, indicating that CsMYB30 and CsJAZ4/6 can interact in yeast cells (Figure 9A,B).

CsMYB30 and CsJAZ4/6 interactions were further detected in tobacco leaves by bimolecular fluorescence complementation (BiFC) experiments. The results showed that the co-transformation of nYFP-CsJAZ4+cYFP with nYFP-CsJAZ6+cYFP and nYFP+CsMYB30-cYFP combinations could not produce fluorescence signals in tobacco leaves, while in the co-transformation of nYFP-CsMYB30+CsJAZ4-cYFP, nYFP-CsMYB30+CsJAZ6-cYFP combination cells, fluorescent signals could be observed at the position of the nucleus, and the fluorescent signals overlapped with those emitted by the Nuclear Marker, indicating that CsMYB30 and CsJAZ4/6 could interact with each other (Figure 9C).

The interaction between CsMYB30 on CsJAZ4/6 was further verified in plants by luciferase complementation imaging (LCI) experiments. The results showed that tobacco detected fluorescent signals only in the nLuc-CsMYB30+CsJAZ4-cLuc and nLuc-CsMYB30+CsJAZ6-cLuc co-transformed regions, and no fluorescent signals were detected in the transformed regions of the other three plasmid combinations, suggesting that CsMYB30 and CsJAZ4 and CsMYB30 and CsJAZ6 can have an interaction. When MeJA was sprayed exogenously, the fluorescence signals were detected to be weakened in the co-transformed regions of nLuc-CsMYB30+CsJAZ4-cLuc and nLuc-CsMYB30+CsJAZ6-cLuc (Figure 9D,E).

These results indicate that an interaction exists between CsMYB30 and CsJAZ4/6, while MeJA attenuated the interaction relationship between CsMYB30 and CsJAZ4/6 proteins.

## 3. Discussion

RNA-seq analysis identified *lnc015013* as a key regulator under low-temperature stress. LncRNAs are widely involved in plant stress responses [20]. By mining RNA-seq data [21], we confirmed that *lnc015013* is a non-coding transcript with the highest expression in roots. Under cold and MeJA co-treatment, *lnc015013* was up-regulated 2.59-fold, *CsMYB30* 16.44-fold, *CsJAZ4* 455.05-fold and *CsJAZ6* 66.37-fold, validating the RNA-seq accuracy and supporting the role of *lnc015013* in cold resistance.

The positive function of *lnc015013* was verified by heterologous expression in Arabidopsis, as well as by transient silencing and overexpression in tea plants. Exogenous MeJA further enhanced this effect, highlighting the importance of JA signaling. Because lncRNAs are not translated [22], they act through interactions with DNA, RNA or proteins. For instance, rice lncRNA tcon_00021861 acts as an miRNA sponge [23]; Arabidopsis APOLO interacts with *WRKY42* [24]; wheat VAS recruits the RF2b-RF2a complex [25]; and COOLAIR forms nuclear condensates with FRI [26]. Although the exact mechanism of lnc015013 remains to be fully characterized, our evidence indicates that it indirectly promotes cold tolerance by up-regulating *CsMYB30*, *CsJAZ4* and *CsJAZ6*.

Further analysis showed that CsMYB30 directly binds the promoters of *CsJAZ4/6* and represses their expression, while *CsJAZ4/6* in turn inhibit JA signaling by interacting with CsMYB30, thereby reducing cold tolerance. Exogenous MeJA weakens this interaction, relieving the repression and enhancing JA pathway activity. The apparent paradox—that *lnc015013* up-regulates both a positive regulator (*CsMYB30*) and negative regulators (*CsJAZ4/6*)—is resolved by post-translational regulation. Although *lnc015013* increases *CsJAZ4/6* transcript levels, under cold stress and exogenous MeJA treatment, the CsMYB30–CsJAZ4/6 interaction is significantly weakened (Figure 9D,E), and JAZ proteins are likely degraded via the COI1-mediated ubiquitination pathway. Consequently, the functional output at the protein level is dominated by the release of CsMYB30 from inhibition, allowing it to activate JA biosynthesis and downstream cold-responsive genes. Thus, the elevated CsJAZ4/6 transcripts do not compromise cold tolerance. This is consistent with known JA-mediated JAZ protein degradation [27]. For example, JAZ repressors interact with ABI3 to suppress ABA signaling [28], and MdJAZ1 interferes with MdTRB1–MdMYB9 to inhibit anthocyanin biosynthesis [29]. Our previous work showed that CsMYB30 is a positive cold regulator, and overexpression of DgMYB2 improves chrysanthemum cold tolerance [30]. JAZ proteins are well-established negative regulators [31]; cold stress increases JA content, leading to JAZ ubiquitination and release of ICE1/DREB1/CBF, which activate downstream COR genes. In our study, CsJAZ4/6 restrict CsMYB30 under cold, whereas MeJA alleviates this restriction, allowing CsMYB30 to exert its full protective function.

A seeming paradox is that *lnc015013* transcriptionally up-regulates both *CsMYB30* (a positive regulator) and *CsJAZ4/6* (negative regulators) while CsMYB30 represses the *CsJAZ4/6* promoter. This is not a contradiction but a reflection of multi-layer regulation. This design—co-induction of a positive regulator and its own repressor—is a well-established feature of JA signaling, exemplified by the MYC2-JAZ negative feedback loop [32]. LncRNAs can act as regulatory hubs that influence multiple target genes through independent mechanisms [22]. In our system, *lnc015013* likely activates *CsMYB30* and *CsJAZ4/6* via independent paths; the direct repression of the *CsJAZ4/6* promoter by CsMYB30 is a separate, parallel input. The net increase in *CsJAZ4/6* transcripts upon *lnc015013* overexpression simply indicates that the activation path outweighs the repression effect. Moreover, under cold stress and exogenous MeJA, JAZ proteins are rapidly degraded via the COI1-mediated ubiquitination pathway [33,34], and the CsMYB30–CsJAZ4/6 interaction is significantly weakened (Figure 9D,E). Consequently, the functional output is dominated by the release of CsMYB30 from inhibition, leading to enhanced JA biosynthesis and cold tolerance.

Nevertheless, whether *lnc015013* directly binds DNA, RNA or proteins remains unknown. We propose several non-exclusive mechanisms for future investigation: (i) *lnc015013* may act in cis by influencing the chromatin state or transcription of its neighboring genes; (ii) it may function as a molecular sponge for miRNAs that target *CsMYB30* or *CsJAZ4/6* mRNAs; or (iii) it could interact with an unknown RNA-binding protein that modulates the stability or translational efficiency of these transcripts. Testing these hypotheses will require a combination of RNA immunoprecipitation (RIP), RNA pull-down, chromatin isolation by RNA purification (ChIRP), and reporter assays. Future work using these approaches will clarify the mode of action of lnc015013 and complete the mechanistic understanding of the lnc015013-CsMYB30-CsJAZ4/6 module in JA-mediated cold tolerance.

We note that JA quantification in this study was performed by ELISA, which is less precise than LC-MS-based methods. The relative changes in JA content between treatment groups were consistent across independent experiments and correlated with phenotypic observations, supporting the qualitative conclusion. Future work employing LC-MS will provide more accurate absolute quantitation of jasmonates and their metabolites.

In summary, this study elucidates the lnc015013–CsMYB30–CsJAZ4/6 regulatory module that integrates JA signaling to enhance cold tolerance in tea plants, deepening our understanding of lncRNA function in stress responses and providing a theoretical basis for cold-resistance breeding.

## 4. Materials and Methods

### 4.1. Experimental Materials and Growth

The annual “Longjing 43” cuttings used in this experiment were purchased from Jiangsu Bocha Agricultural Science and Technology Development Co., Ltd. (Nanjing, China) and placed in an artificial climatic chamber with day and night temperatures of 25 °C/18 °C, a photoperiod of 14 h/10 h, a light intensity of 100 μmol·m^−2^·s^−1^, and a relative humidity of 65% for soil cultivation to slow down the seedlings. The Arabidopsis plants and tobacco used in this experiment were obtained from the Laboratory of Tea Science, College of Horticulture, Nanjing Agricultural University. Both Arabidopsis (*Arabidopsis thaliana*) and tobacco (*Nicotiana benthamiana*) model plants were grown in an artificial climate chamber under controlled conditions (22 °C:16 °C, 16 h:8 h, light:dark photoperiod, a light intensity of 120 μmol·m^−2^·s^−1^, and a relative humidity of 55%).

### 4.2. RNA-Seq Sequencing and Library Construction

The library construction and sequencing of RNA-seq were entrusted to Guangzhou Kidio Biotechnology Co. (Guangzhou, China). The total RNA of the samples was first extracted, and then the ribosomal RNA (rRNA) was removed, retaining all the coding RNA and non-coding RNA. The RNA was fragmented to 200–500 nt and used as a template to synthesize the first strand of cDNA using six-base random primers (Random hexamers, Qiagen, Hilden, Germany). The second strand of cDNA was synthesized by adding dNTPs, RNase H and DNA polymerase I. The library was purified and built using a QiaQuick PCR kit (Qiagen, Hilden, Germany) and sequenced using Illumina HiSeq^TM^ 4000 (Illumina, Inc., San Diego, CA, USA).

Bioinformatics analysis of RNA-seq data: Raw reads were processed using fastp v0.23.4 (https://github.com/OpenGene/fastp, accessed on 8 May 2026) to remove adapters, reads with >10% ambiguous bases (N), and low-quality reads. Clean reads were evaluated for Q20, Q30 and GC content. Clean reads were aligned to the tea plant reference genome (TPIA) using hisat2, and transcripts were assembled with Stringtie. For lncRNA identification, transcripts ≥200 bp were assessed for coding potential using CPC and CNCI. The intersection of transcripts predicted as non-coding by both tools was retained as reliable lncRNAs, which were then classified according to their genomic position relative to protein-coding genes. Expression levels (FPKM) were calculated, and differential expression analysis was performed using DESeq2 with thresholds of |log2(fold change)| ≥ 1 and an adjusted *p*-value < 0.05 (Benjamini–Hochberg FDR correction).

Targets of lncRNAs were predicted as follows: (i) cis targets, genes located within 10 kb upstream or downstream of an lncRNA; (ii) trans targets, identified by expression correlation analysis between lncRNAs and mRNAs across samples; (iii) antisense targets, predicted using RNAplex based on the minimum free energy of base pairing. Venn diagrams were generated using Venny2.1. lncRNA–mRNA network graphs were constructed using Gephi (version 0.10.1). GO and KEGG enrichment analyses of target genes were performed using the Omicshare platform (version 3.0). Subcellular localization of lncRNAs was predicted using lncLocator (version 2.0), and RNA–protein interactions were predicted using RPIseq (version 1.0).

### 4.3. Tea Plant Materials and Low-Temperature Treatments

Tea seedlings with consistent and good growth were treated with 4 °C + H_2_O (C), MeJA (M), and 4 °C + MeJA (CM), and normal-growing tea seedlings were used as control (CK), with a MeJA concentration of 100 μmol·L^−1^. Both water and MeJA were sprayed until the leaves were wetted, and the treatments were carried out for 4 days, with three sets of biological replicates for each treatment. One bud and two leaves were frozen in liquid nitrogen and stored in a refrigerator at −80 °C.

WT, EV, and transgenic Arabidopsis seeds were grown in seeding culture. After the seedlings were grown to about 4 weeks of age, they were subjected to the following four treatments: (1) CK (normal growth conditions, sprayed with water); (2) MeJA (sprayed with 30 μM MeJA, grown under normal conditions); (3) cold (sprayed with water and placed at 4 °C for 24 h); (4) cold+MeJA (sprayed with 30 μM MeJA and placed at 4 °C for 24 h). Three biological replicates (each replicate containing 10 seedlings) were performed for each genotype and treatment. The plants were kept under a normal photoperiod and humidity, as described in Section 4.1.

The 100 μM MeJA for tea plants was selected based on our previous study [9], which demonstrated that this concentration effectively scavenges reactive oxygen species and maintains membrane stability under cold stress. For *Arabidopsis*, a lower concentration of 30 μM was chosen because *Arabidopsis* is highly sensitive to exogenous jasmonates; our preliminary experiments showed that 30 μM induced cold-related responses without causing growth inhibition or phytotoxicity, whereas higher concentrations (≥50 μM) caused visible root growth inhibition and leaf damage.

### 4.4. Tea Plant Transient Silence

The Sfold website (https://sfold.wadsworth.org/cgi-bin/soligo.pl, accessed on 8 May 2026) was used to design antisense oligonucleotide (AsODN) sequence primers for lncRNAs, and sense oligonucleotides (Sense) sequence primers were used as controls. Three primers were designed for each lncRNA, and the primers were synthesized by Nanjing Kengke Biotechnology Co. (Nanjing, China). The primers were dissolved in sterilized water and diluted to 20 μM. After mixing the three primers for each lncRNA, 1 mL of the 20 μM mixed primer was injected into tea plant leaves using a needleless syringe and incubated normally for 24 h. Then, the leaves were taken to liquid nitrogen, frozen, and stored in a refrigerator at −80 °C. Three independent biological replicates (each using leaves from different tea plants) were performed.

### 4.5. Tea Plant Transient Overexpression

The lncRNA was inserted into the PBI121-GFP vector and transferred to the host bacterium GV3101; the PBI121-GFP empty vector was used as a control. Then, the bacterial solution was washed, resuspended in MES suspension, and injected into mature leaves of tea branches. After incubation for 72 h, the samples were frozen in liquid nitrogen for gene expression and physiological analysis. At least 3 biological replicates were included.

### 4.6. Heterologous Expression in Arabidopsis thaliana

Cloning and plasmid construction were performed following standard molecular biology protocols. The cDNA of the terminal bud and first leaf of “Longjing 43” was used as a template for cloning. The full-length sequence of lncRNA was amplified by PCR and inserted into a pEASY-Blunt simple zero cloning vector. The sequence of the cloned lncRNA was confirmed by sequencing. The clone was then cloned into the expression vector PBI121 using the Gateway LR clonase system. The expression vector for the target gene PBI121-lncRNA was transferred into the expression host strain GV3101. Primer sequences are shown in Table S1. Recombinant PBI121-lncRNA plasmid was chemically transformed into GV3101. Agrobacterium-mediated transformation was performed using wild-type *Arabidopsis thaliana* (Col-0). Heterologous transformation of CsMYB30, CsJAZ4/6 was performed as described above. For each transgenic line, at least three independent T2 or T3 lines were used for phenotypic analysis, and each experiment was repeated three times with similar results. Note that in this study, the term “heterologous expression” is used because the transgenes originate from tea plants (*Camellia sinensis*) and are expressed in *Arabidopsis thaliana*; we did not independently verify high-level overexpression of these transgenes beyond the mRNA level measured by qRT-PCR.

### 4.7. Determination of Malondialdehyde, Proline and JA in Arabidopsis thaliana

The malondialdehyde content of *Arabidopsis thaliana* samples was determined by using a malondialdehyde (MDA) content assay kit (Beijing Solepol Technology Co., Ltd., Beijing, China) according to the operational procedures. The endogenous JA content of *Arabidopsis thaliana* and tea plant was determined by weighing 1 g of tea plant frozen sample tissue, adding 9 mL of PBS buffer of pH 7.2–7.4, grinding the sample well, centrifuging the sample for 20 min at 4 °C, 3000 rpm/min, and collecting the supernatant. The JA content of the samples was determined using a plant jasmonic acid (JA) ELISA kit (Jiangsu Enzyme Immunity Industry Co., Ltd., Yancheng, China). Three biological replicates were measured for each sample, and each measurement was performed in triplicate.

### 4.8. RNA Extraction and qRT-PCR Analysis

Sample RNA was extracted using a FastPure Plant Total RNA Isolation Kit (Nanjing Novozymes Bioscience & Technology Co., Ltd., Nanjing, China), and RNA was reverse-transcribed into cDNA using the HiScript III RT SuperMix for qPCR (+gDNA wiper) reagent (Nanjing Novozymes Bioscience & Technology Co., Ltd., Nanjing, China). Primers for fluorescent quantitative PCR were designed using Primer Premier 5.0, as shown in Table S1. The primers were synthesized by Nanjing Prime Biotechnology Co. The sample cDNA was used as a template for amplification and quantitative gene expression analysis using ChamQ SYBR qPCR Master Mix (Nanjing Novozymes BioScience and Technology Co., Ltd., Nanjing, China) on a fluorescent quantitative PCR instrument (Quantitative real-time PCR CFX 96, BioRad, Hercules, CA, USA). The reaction program was 95 °C, 30 s, 95 °C, 10 s, 60 °C, 30 s, and cycling 40 times, and gene expression was calculated by the 2^−∆∆Ct^ method. Three biological replicates were used for each treatment, and each sample was technically repeated three times. The 2^−∆∆Ct^ method was applied.

qRT-PCR normalization: We then added the internal reference genes used: for tea plants, β-actin (NCBI accession number XM_082887030.1), and for Arabidopsis, ACT2 (actin 2, Gene ID 821411). We also clarify that the 2^−ΔΔCt^ method was applied with three biological replicates and three technical repeats per sample. 

### 4.9. Yeast Two-Hybrid (Y2H) Test

CsJAZ4/6 was cloned into the pGBKT7 vector as bait for Y2H screening. The CsMYB30 coding sequence was cloned into the pGADT7 vector as prey (primers are listed in Table S1). Haploid Saccharomyces cerevisiae Y2H from the Matchmaker two-hybrid system (Clontech, San Jose, CA, USA) was transformed with bait and prey vectors according to the kit instructions. The transformed yeast clones were screened on SD/-Leu/-Trp medium and then transferred to SD/-Leu/-Trp/-His/-Ade medium. The interaction of pGBKT7-CsJAZ4/CsJAZ6 with pGADT7-CsMYB30 was assayed, and the association of pGBKT7-CsJAZ4/CsJAZ6 with pGADT7 served as a negative control. The experiment was independently performed three times with similar results.

### 4.10. Bimolecular Fluorescence Complementation (BiFC) Assay

The CsJAZ4/6 coding sequences were inserted into 35S::pSPYNEnYFP, and the CsMYB30 sequence was inserted into 35S::pSPYCEcYFP. bifc assays were performed using 5-week-old benthamiana leaves following the fluorescence detection method of Gookin and Assmann21. In benthamiana cells, co-expression of CsJAZ4-nYFP and CsJAZ6-nYFP with CsMYB30-cYFP promoted protein interactions leading to the formation of fluorescent complexes. A co-laser confocal microscope (LSM 900, Zeiss, Jena, Germany) was used. At least ten tobacco leaf cells were examined per combination, and the experiment was repeated three times with independent leaf infiltrations.

### 4.11. Split Luciferase Complementation Assay

CsJAZ4/6, which lacks a stop codon, was cloned into pCAMBIA1300nLUC, and CsMYB30 was cloned into pCAMBIA1300-cLUC. The split luciferase complementation assay involved transient expression in *N. benthamiana* leaves by agroinfiltration. In leaves co-expressing different structures, luciferase activity was assayed by applying 1 mM d-luciferin, followed by a 5 min dark incubation before imaging. A CCD camera (Tanon 5200, Shanghai Tanon Life Science Co., Ltd., Shanghai, China) was used to detect luciferase activity and acquire images. Ten leaves of five 1-month-old benthamiana plants were used as biological replicates.

### 4.12. Yeast Single-Hybrid Experiment (Y1H)

Y1H assays were performed to determine whether CsMYB30 interacts with DNA sequences within 2 kb upstream of the target gene ATG. The full-length CDS of CsMYB30 was transferred on the pGADT7 vector as a prey expression construct. On the decoy expression vector, a DNA fragment of approximately 2 kb upstream from the TG of CsJAZ4/6 was amplified, and the fragments were fused into the pHIS2 vector. The primers used are shown in Supplemental Table S1. The bait-eating constructs and bait constructs were co-transformed into yeast using the One-Hybrid System protocol (TaKaRa, Tokyo, Japan). A pGADT7 empty vector was used as a negative control, and p53 was used as a positive control. The yeast was cultured on 28 °C SD-Trp/-His/-Leu selective medium for 2–3 days. The growth of yeast colonies was observed, recorded, and photographed. Three independent yeast transformations were performed for each construct combination.

### 4.13. Dual Luciferase Assay

In the dual luciferase reporter gene assay, a 2000 bp CsJAZ4/6 promoter sequence and a full-length CsMYB30 CDS were inserted into pGreen II 0800LUC and pGreen II 62-SK vectors, respectively, to generate the reporter plasmid and effector plasmid. The pGreenII 62-SK empty vector was used as a negative control. The recombinant plasmids were transformed into GV3101 cells using the heat shock method. The transformed Agrobacterium strains were cultured to OD600 = 1.0, and the suspension was incubated for 2 h. Tobacco leaves with good growth conditions after 5–6 weeks were selected for injection. After 2–3 days of incubation in the dark, a dual luciferase reporter assay system was used, and binding activity was determined by the LUC/REN ratio. Six biological replicates were used for each experiment. Statistical analysis was performed to report the mean and standard deviation of the data, and statistical significance was determined by a one-way ANOVA test using SPSS. A *t*-test was used to determine significant differences between data.

### 4.14. Statistical Analysis

Each experiment consisted of three independent biological replicates. The experimental data were organized using the software Excel 2020, analyzed for significance using the software IBM SPSS Statistics 17.0, and plotted using the software GraphPad 8.0.1. Different lowercase letters indicate significant differences at the *p* < 0.05 level, and different asterisks mark significance as follows: *p* < 0.05, *p* < 0.01, *p* < 0.001. All data are presented as mean ± standard deviation (SD). Error bars in figures represent SD.

## 5. Conclusions

**Figure 10:**
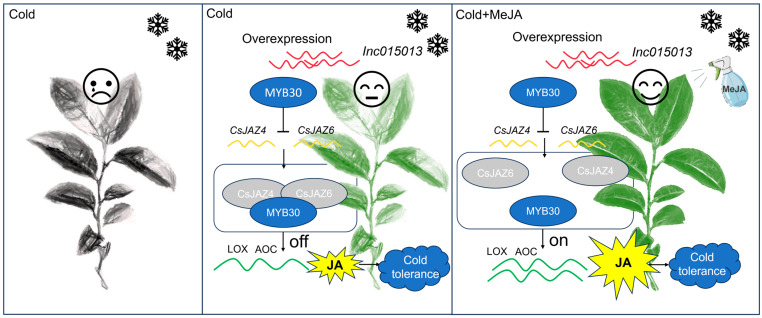
lnc015013-CsMYB30-CsJAZ4/6 module mediates jasmonic acid signaling pathway and exogenous MeJA synergistically enhances the pattern of cold resistance in tea plants.

In conclusion, this study elucidates the molecular mechanisms underlying lncRNA involvement in JA-mediated regulation of the cold stress response in tea plants, thereby filling critical knowledge gaps (Figure 10). It offers novel insights into the function of plant non-coding RNAs in stress responses. Our findings corroborate previous research on the role of JA in plant chilling resistance and further establish a new role for lncRNAs in this process. While this study has uncovered the role of the lnc015013-CsMYB30-CsJAZ4/6 module in the cold stress response of tea plants, there remain many unexplored areas worthy of further investigation. For instance, the interaction network between lnc015013 and other genes, the upstream and downstream regulators within the JA signaling pathway, and the expression patterns of these genes across different tea plant cultivars all merit additional research.

## Data Availability

The original contributions presented in this study are included in the article/Supplementary Materials. Further inquiries can be directed to the corresponding author.

## Supplementary Materials

The following supporting information can be downloaded at https://www.mdpi.com/article/10.3390/ijms27114776/s1.

## Abbreviations

The following abbreviations are used in this manuscript:

AsODN	antisense oligonucleotide
BiFC	bimolecular fluorescence complementation
EV	empty vector
Fv/Fm	maximum photochemical efficiency of photosystem II
GO	Gene Ontology
JA	jasmonic acid
JAZ	jasmonate ZIM-domain
KEGG	Kyoto Encyclopedia of Genes and Genomes
LCI	luciferase complementation imaging
lncRNA	long non-coding RNA
MDA	malondialdehyde
MeJA	methyl jasmonate
MYB	myeloblastosis (transcription factor)
NBT	nitroblue tetrazolium
OE	overexpression
PCR	polymerase chain reaction
POD	peroxidase
qPCR	quantitative polymerase chain reaction
qRT-PCR	quantitative real-time polymerase chain reaction
RNA-seq	RNA sequencing
ROS	reactive oxygen species
SOD	superoxide dismutase
WT	wild type

## References

[1] Zaman S., Hassan S.S.U., Ding Z. (2022). The Role of Calmodulin Binding Transcription Activator in Plants under Different Stressors: Physiological, Biochemical, Molecular Mechanisms of *Camellia sinensis* and Its Current Progress of CAMTAs. Bioengineering.

[2] Gusain S., Joshi S., Joshi R. (2023). Sensing, signalling, and regulatory mechanism of cold-stress tolerance in plants. Plant Physiol. Biochem..

[3] Zhao M., Zhang N., Gao T., Jin J., Jing T., Wang J., Wu Y., Wan X., Schwab W., Song C. (2019). Sesquiterpene glucosylation mediated by glucosyltransferase UGT91Q2 is involved in the modulation of cold stress tolerance in tea plants. New Phytol..

[4] An J., Wang X., Zhang X., You C., Hao Y. (2020). Apple B-box protein BBX37 regulates jasmonic acid mediated cold tolerance through the JAZ-BBX37-ICE1-CBF pathway and undergoes MIEL1-mediated ubiquitination and degradation. New Phytol..

[5] Hu Y., Jiang Y., Han X., Wang H., Pan J., Yu D. (2017). Jasmonate regulates leaf senescence and tolerance to cold stress: Crosstalk with other phytohormones. J. Exp. Bot..

[6] Huang H., Liu B., Liu L., Song S. (2017). Jasmonate action in plant growth and development. J. Exp. Bot..

[7] Wang Y., Mostafa S., Zeng W., Jin B. (2021). Function and Mechanism of Jasmonic Acid in Plant Responses to Abiotic and Biotic Stresses. Int. J. Mol. Sci..

[8] Wang L., Chen H., Chen G., Luo G., Shen X., Ouyang B., Bie Z. (2023). Transcription factor SlWRKY50 enhances cold tolerance in tomato by activating the jasmonic acid signaling. Plant Physiol..

[9] Han Z., Zhang C., Zhang H., Duan Y., Zou Z., Zhou L., Zhu X., Fang W., Ma Y. (2022). CsMYB Transcription Factors Participate in Jasmonic Acid Signal Transduction in Response to Cold Stress in Tea Plant (*Camellia sinensis*). Plants.

[10] Palos K., Yu L., E Railey C., Dittrich A.C.N., Nelson A.D.L. (2023). Linking discoveries, mechanisms, and technologies to develop a clearer perspective on plant long noncoding RNAs. Plant Cell.

[11] Choi S.-W., Kim H.-W., Nam J.-W. (2019). The small peptide world in long noncoding RNAs. Brief. Bioinform..

[12] Zhang L., Wang M., Li N., Wang H., Qiu P., Pei L., Xu Z., Wang T., Gao E., Liu J. (2017). Long noncoding RNAs involve in resistance to *Verticillium dahliae*, a fungal disease in cotton. Plant Biotechnol. J..

[13] Hong Y., Zhang Y., Cui J., Meng J., Chen Y., Zhang C., Yang J., Luan Y. (2022). The lncRNA39896–miR166b–*HDZs* module affects tomato resistance to *Phytophthora infestans*. J. Integr. Plant Biol..

[14] Jha U.C., Nayyar H., Jha R., Khurshid M., Zhou M., Mantri N., Siddique K.H.M. (2020). Long non-coding RNAs: Emerging players regulating plant abiotic stress response and adaptation. BMC Plant Biol..

[15] Chen X., Jiang X., Niu F., Sun X., Hu Z., Gao F., Zhang H., Jiang Q. (2023). Overexpression of *lncRNA77580* Regulates Drought and Salinity Stress Responses in Soybean. Plants.

[16] Cui J., Jiang N., Meng J., Yang G., Liu W., Zhou X., Ma N., Hou X., Luan Y. (2018). LncRNA33732-respiratory burst oxidase module associated with WRKY1 in tomato- *Phytophthora infestans* interactions. Plant J..

[17] Zhang M., Yang H., Chen Z., Hu X., Wu T., Liu W. (2021). Long Noncoding RNA X-Inactive-Specific Transcript Promotes the Secretion of Inflammatory Cytokines in LPS Stimulated Astrocyte Cell Via Sponging miR-29c-3p and Regulating Nuclear Factor of Activated T cell 5 Expression. Front. Endocrinol..

[18] Song Y., Li L., Yang Z., Zhao G., Zhang X., Wang L., Zheng L., Zhuo F., Yin H., Ge X. (2019). Target of Rapamycin (TOR) Regulates the Expression of lncRNAs in Response to Abiotic Stresses in Cotton. Front. Genet..

[19] Böhmdorfer G., Wierzbicki A.T. (2015). Control of Chromatin Structure by Long Noncoding RNA. Trends Cell Biol..

[20] Sun H.X., Chua N.H. (2019). Bioinformatics Approaches to Studying Plant Long Noncoding RNAs (lncRNAs): Identification and Functional Interpretation of lncRNAs from RNA-Seq Data Sets. Methods Mol. Biol..

[21] Kang C., Liu Z. (2019). An Easy-to-Follow Pipeline for Long Noncoding RNA Identification: A Case Study in Diploid Strawberry *Fragaria vesca*. Methods Mol. Biol..

[22] Mattick J.S., Amaral P.P., Carninci P., Carpenter S., Chang H.Y., Chen L.-L., Chen R., Dean C., Dinger M.E., Fitzgerald K.A. (2023). Long non-coding RNAs: Definitions, functions, challenges and recommendations. Nat. Rev. Mol. Cell Biol..

[23] Mao Y., Xu J., Wang Q., Li G., Tang X., Liu T., Feng X., Wu F., Li M., Xie W. (2021). A natural antisense transcript acts as a negative regulator for the maize drought stress response gene *ZmNAC48*. J. Exp. Bot..

[24] Moison M., Pacheco J.M., Lucero L., Fonouni-Farde C., Rodríguez-Melo J., Mansilla N., Christ A., Bazin J., Benhamed M., Ibañez F. (2021). The lncRNA APOLO interacts with the transcription factor WRKY42 to trigger root hair cell expansion in response to cold. Mol. Plant.

[25] Xu S., Dong Q., Deng M., Lin D., Xiao J., Cheng P., Xing L., Niu Y., Gao C., Zhang W. (2021). The vernalization-induced long non-coding RNA VAS functions with the transcription factor TaRF2b to promote TaVRN1 expression for flowering in hexaploid wheat. Mol. Plant.

[26] Zhu P., Lister C., Dean C. (2021). Cold-induced Arabidopsis FRIGIDA nuclear condensates for FLC repression. Nature.

[27] Major I.T., Yoshida Y., Campos M.L., Kapali G., Xin X., Sugimoto K., Ferreira D.d.O., He S.Y., Howe G.A. (2017). Regulation of growth–defense balance by the JASMONATE ZIM-DOMAIN (JAZ)-MYC transcriptional module. New Phytol..

[28] Pan J., Hu Y., Wang H., Guo Q., Chen Y., Howe G.A., Yu D. (2020). Molecular Mechanism Underlying the Synergetic Effect of Jasmonate on Abscisic Acid Signaling during Seed Germination in Arabidopsis. Plant Cell.

[29] An J., Xu R., Liu X., Zhang J., Wang X., You C., Hao Y. (2021). Jasmonate induces biosynthesis of anthocyanin and proanthocyanidin in apple by mediating the JAZ1–TRB1–MYB9 complex. Plant J..

[30] Yang X., Luo Y., Bai H., Li X., Tang S., Liao X., Zhang L., Liu Q. (2022). DgMYB2 improves cold resistance in chrysanthemum by directly targeting *DgGPX1*. Hortic. Res..

[31] Hu T., Zeng H., Hu Z., Qv X., Chen G. (2013). Overexpression of the Tomato 13-Lipoxygenase Gene *TomloxD* Increases Generation of Endogenous Jasmonic Acid and Resistance to *Cladosporium fulvum* and High Temperature. Plant Mol. Biol. Report..

[32] Chico J.M., Chini A., Fonseca S., Solano R. (2008). JAZ repressors set the rhythm in jasmonate signaling. Curr. Opin. Plant Biol..

[33] Sheard L.B., Tan X., Mao H., Withers J., Ben-Nissan G., Hinds T.R., Kobayashi Y., Hsu F.-F., Sharon M., Browse J. (2010). Jasmonate perception by inositol-phosphate-potentiated COI1-JAZ co-receptor. Nature.

[34] Thines B., Katsir L., Melotto M., Niu Y., Mandaokar A., Liu G., Nomura K., He S.Y., Howe G.A., Browse J. (2007). JAZ repressor proteins are targets of the SCF(COI1) complex during jasmonate signalling. Nature.

