# Supplementary material for: lnc015013-CsMYB30-CsJAZ4/6 Module Co-Regulates JA Synthesis and Enhances Cold Hardiness in Tea Plants
Source: Int J Mol Sci. 2026 May 26;27(11):4776. doi: 10.3390/ijms27114776 (PMC13257133; doi:10.3390/ijms27114776)
Supplement: Supplementary file 1 [file ijms-27-04776-s001.zip › ijms-4272225-supplementary.pdf]

Supplemental Table S1: Primer sequences.

Experiment name	Gene name	Primer sequence 5'-3'
q-PCR	lncRNA015013-F-DL	GCTCCGACGCTCCTTACCAC
	lncRNA015013- R-DL	ACAGCCGTGACGTTCCAAGTAC
	Cs*JAZ4*- F-DL	GACTGTGATTGCTTCTGGCTCT
	Cs*JAZ4*- R-DL	GACAACGCGAGTTCTGGATTTA
	Cs*JAZ6*- F-DL	GGCTCTAGCCTGAAGCTCTGTA
	Cs*JAZ6*- R-DL	ACAGCCGTGACGTTCCAAGTAC
	β-actin-F	TTGGCATCGTTGAGGGTCT
	β-actin-R	CAGTGGGAACACGGAAAGC
Gene clone	lncRNA015013-F	GGGAATCTAAACCCTAT
	lncRNA015013-R	CTAGATCATGATGGATGTGAACTGC
	sODN-*lnc015013*-1	ACTATAACTTTAGGGAAGCC
	sODN- *lnc015013*-2	AGCAACCGAGAGGATAGAAG
	sODN- *lnc015013*-3	ACCCATTCCTACGGCAAAGT
	AsODN- *lnc015013*-1	TGATATTGAAATCCCTTCGG
	AsODN- *lnc015013*-2	TCGTTGGCTCTCCTATCTTC
	AsODN- *lnc015013*-3	TGGGTAAGGATGCCGTTTCA
	Cs*JAZ4*-CDS-F	ATGGCATCGAGATCAGCTGTTGAAC
	Cs*JAZ4*-CDS-R	ATCTTCATATCTTGATGTATTCTTC
	Cs*JAZ6*-CDS-F	ATGAAAATGAGGAGGAATTGTAACT
	Cs*JAZ6*-CDS-R	GTGATTATAAGGAGATGTTGCTTGA
Promoter clone	Cs*JAZ4*-2000-F	AATAAGGGTGGGTTTTTGAGTC
	Cs*JAZ4*-2000-R	TTTGATGTTCAACTCTTTTTCTCTT
	Cs*JAZ6*-2000-F	CACATGGACGCCCAATTTCTA
	Cs*JAZ6*-2000-R	TATGTTTCTTCAGTATATATCTCTT
Y2H experiment	Cs*JAZ4*-BD-F	AGGCCGAATTCCCGGGGATCCATGGCATCG AGATCAGCTGTTGAAC
	Cs*JAZ4*-BD-R	CCGCTGCAGGTCGACGGATCCATCTTCATAT CTTGATGTATTCTTC

Table (continued)

Experiment name	Gene name	Primer sequence 5’-3’
	Cs*JAZ6*-BD-F	AGGCCGAATTCCCGGGGATCCATGAAAATG AGGAGGAATTGTAACT
	Cs*JAZ6*-BD-R	CCGCT GCAGGTCGACGGATCCGTGATTATAAGGAG ATGTTGCTTGA
	Cs*MYB30*-AD	GCCATGGAGGCCAGTGAATTCATGTCTTCTA GTTTGACCAAGAGTGC
	Cs*MYB30*-AD	CAGCTCGAGCTCGATGGATCCTAAAACGAC CCATGATTCATCCA
Bifc experiment	Cs*MYB30*-C/N-Fbifc	GAGCTCGGTACCCGGGGATCCATGTCTTCTA GTTTGACCAAGAGTGC
	Cs*MYB30*-C/N-Rbifc	GAGCTCGGTACCCGGGGATCCTAAAACGAC CCATGATTCATCCA
	Cs*JAZ4*-C/N-Fbifc	GAGCTCGGTACCCGGGGATCCATGGCATCG AGATCAGCTGTTGAAC
	Cs*JAZ4*-C/N-Rbifc	GAGCTCGGTACCCGGGGATCCATCTTCATAT CTTGATGTATTCTTC
	Cs*JAZ6*-C/N-Fbifc	GAGCTCGGTACCCGGGGATCCATGAAAATG AGGAGGAATTGTAACT
	Cs*JAZ6*-C/N-Rbifc	GAGCTCGGTACCCGGGGATCCGTGATTATAA GGAGATGTTGCTTGA
LCI experiment	Cs*JAZ4*-cluc-F	CCGGGGCGGTACCCGGGATCCATGGCATCG AGATCAGCTGTTGAAC
	Cs*JAZ4*-cluc-R	GCCGGGCCCTCTAGAGGATCCATCTTCATAT CTTGATGTATTCTTC
	Cs*JAZ6*-cluc-F	CCGGGGCGGTACCCGGGATCCATGAAAATG AGGAGGAATTGTAACT
	Cs*JAZ6*-cluc-R	GCCGGGCCCTCTAGAGGATCCGTGATTATAA GGAGATGTTGCTTGA

Table (continued)

Experiment name	Gene name	Primer sequence 5’-3’
LUC experiment	Cs*MYB30*-nluc-F	CGAGCTCGGTACCCGGGATCCATGTCTTCTAGTTTGACCAAGAGTGC
	Cs*MYB30*-nluc-R	GCCGGGCCCTCTAGAGGATCCTAAAACGACCCATGATTCATCCA
	Cs*MYB30*-62-F	TCCCCCGGGCTGCAGGAATTCATGTCTTCTAGTTTGACCAAGAGTGC
	Cs*MYB30*-62-R	GATAAGCTTGATATCGAATTCTAAAACGACCCATGATTCATCCA
	Cs*JAZ4*-0800-F	TTCCTGCAGCCCGGGGGATCCAATAAGGGTGGGTTTTTGAGTC
	Cs*JAZ4*-0800-R	CGCTCTAGAACTAGTGGATCCTTTGATGTTCAACTCTTTTTCTCTT
	Cs*JAZ6*-0800-F	TTCCTGCAGCCCGGGGGATCCCACATGGACGCCCAATTTCTA
	Cs*JAZ6*-0800-R	CGCTCTAGAACTAGTGGATCCTATGTTTCTTCAGTATATATCTCTT
Y1H experiment	Cs*MYB30*-AD-F	GCCATGGAGGCCAGTGAATTCATGTCTTCTAGTTTGACCAAGAGTGC
	Cs*MYB30*-AD-R	CAGCTCGAGCTCGATGGATCCTAAAACGACCCATGATTCATCCA
	Cs*JAZ4*-phis-F	GAATTCCCGGGGAGCTCTGATAAAAAAATAAAAAGATAAAA
	Cs*JAZ4*-phis-R	ATTCGCGAACGCGTGAGCTCGATGTTCAACTCTTTTTCTCTTCTT
	Cs*JAZ6*-phis-F	GAATTCCCGGGGAGCTCTATATAATAAATATTTTTATATTAC
	Cs*JAZ6*-phis-R	ATTCGCGAACGCGTGAGCTCGTTTCTTCAGTATATATCTCTTTCT
